# Protective Effects of Appropriate Amount of Nuts Intake on Childhood Blood Pressure Level: A Cross-Sectional Study

**DOI:** 10.3389/fmed.2021.793672

**Published:** 2022-01-18

**Authors:** Ye Feng, Yang Bi, Xian Tang, Ping Zhang, Jishuang Tong, Xin Peng, Jie Tian, Xiaohua Liang

**Affiliations:** ^1^Department of Clinical Epidemiology and Biostatistics, Children's Hospital of Chongqing Medical University, National Clinical Research Center for Child Health and Disorders, Ministry of Education Key Laboratory of Child Development and Disorders, Chongqing Key Laboratory of Child Health and Nutrition, Chongqing, China; ^2^Chongqing Key Laboratory of Pediatrics, Department of Cardiovascular Medicine, National Clinical Research Center for Child Health and Disorders, Ministry of Education Key Laboratory of Child Development and Disorders, China International Science and Technology Cooperation Base of Child Development and Critical Disorders, Children's Hospital of Chongqing Medical University, Chongqing, China; ^3^School of Computing, Faculty of Engineering and Physical Sciences, University of Leeds, Leeds, United Kingdom

**Keywords:** nuts intake, children, adolescent, blood pressure, childhood hypertension

## Abstract

**Objective:**

Increased blood pressure (BP) is a major risk factor for cardiovascular disease (CVD) in adults. Regular consumption of nuts may improve some BP in adults whereas evidence in children is relatively lacking. This study aimed to determine the efficacy of nuts intake on BP in children.

**Methods:**

Stratified cluster sampling was performed to include a total of 15,268 primary school children aged 6–12 years in urban and rural areas in Southwest China. The daily nuts intake dosage was collected by questionnaires, and generalized linear model (GLM) and logistic regression were used to analyze the relationship between nuts intake and BP.

**Results:**

For the total subjects, 11,130 (72.9%) participants consumed <35 g/day of nuts, 1,145 (7.5%) participants consumed 35 g/day ≤ nut <50 g/day of nuts, 2,053 (13.4%) participants consumed 50~100 g/day of nuts, and 940 (6.2%) participants consumed over 100 g/day of nut. For sex subgroup, 1,074 (13.53%) boys and 979 (13.35%) girls consumed 50~100 g/day of nuts. Compared with the 50~100 g/day of nuts intake group, systolic blood pressure (SBP), diastolic blood pressure (DBP), and mean arterial pressure (MAP) were significantly different in <35 g/day, 35g/day ≤ nut <50 g/day, and >100 g/day nuts intake groups (all *p* < 0.001). The logistic regression showed that compared with the 50~100 g/day group, the other three groups are more likely associated with childhood hypertension (all *p* < 0.001). Therefore, a U-shaped relationship between nuts intake and BP level was identified.

**Conclusions:**

The finding suggests that intake of 50~100 g/day nuts is the recommended dose of nuts intake to control childhood hypertension, as well as for cardioprotection purposes.

## Introduction

Childhood hypertension is a concerning public health issue worldwide (1). It is estimated there is a prevalence of 2–4% childhood hypertension based on the guidelines of the American Academy of Pediatrics (AAP) (2). Recent reports suggest an increased prevalence of childhood hypertension (1, 3), the prevalence of hypertension increased from 4.32 to 7.89% from 2000 to 2015 (4). Childhood hypertension results in significant end-organ damage, which in turn may lead to significant cardiovascular diseases (CVD) later on in life. The end organ damage in the form of cardiac structural changes, which is a consequence of hypertension, can be present in adolescent and early adult life (5–7). Although it is generally accepted that dietary factors, such as salt intake can modulate BP, studies concerning the effects of nut intake on BP are limited, particularly in children and adolescents.

Previous studies revealed that childhood hypertension cannot be simply attributed to genetic factors but was caused by the interaction between environmental factors and genetic factors (8). Among environmental factors, diets play a leading role in BP homeostasis. It has been suggested that adherence to a healthy diet reduces cardiovascular risk by 80–90% (9). Nuts are rich in soluble fiber, antioxidants, and phytosterols, which have beneficial effects on BP (8, 10). In western countries, nuts are consumed as snacks, desserts, or part of a meal (11). In China, nuts are mainly consumed as snacks by children. Nuts contain monounsaturated fatty acid (MUFA) and polyunsaturated fatty acid (PUFA), which were documented to lower BP (12). A study on US adulthood, using the National Health and Nutrition Examination Survey (NHANES) 2005–2010 database (*n* = 14,386) based on 24 h dietary recalls, found that tree nut consumption was linked to lower systolic blood pressure (SBP) (13). Mohan et al. found that a daily intake of nuts improves hypertension risk (8). Some clinical trials showed a decrease in SBP and/or DBP after the consumption of the diet enriched with nuts compared with the control diet (14–16). Moreover, a meta-analysis demonstrated that consumption of 1 ounce (28 g/day) of cashew nut reduces SBP but has no effects on diastolic blood pressure (DBP) for adults (45–56.8 years old) (17). A cross-sectional study enrolled 814 Australian adolescents (13–15 years) revealed that SBP was inversely associated with intakes of omega-3 fatty acids (ω-3 FA) in boys (18). Studies in both adults and children have found the protective effects of nuts on BP, but the evidence from children is rare.

There is a rare study about the dose relationship between nuts and BP, especially from large community-based studies in children. In the present study, we hypothesis that there is a dose-response relationship between nuts intake and BP. Therefore, the current study illustrated the optimal dose of nuts intake in children's BP and provide clear information for dietary recommendations.

## Methods

### Subjects

**Figure 1:**
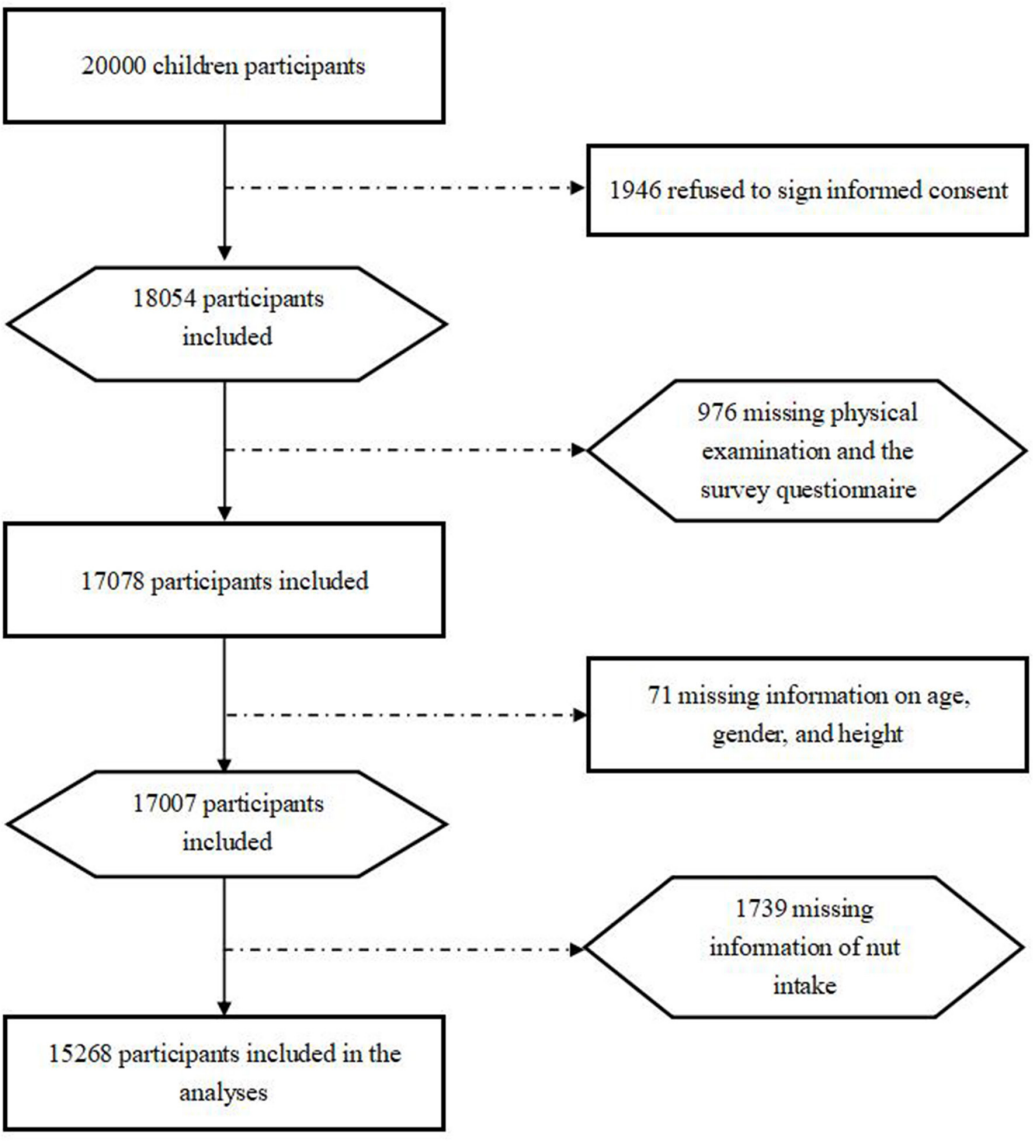
Flow diagram of the selection process of participants.

Details about the study design are available in previous publications (19, 20). In brief, a two-stage stratified cluster sampling was used to include participants from two counties (representing urban and rural areas, respectively) in Chongqing city, China, thereafter, two regions per county were randomly selected. Participants who met all of the following criteria were recruited: (1) aged between 6 and 12 years old in 2014 (19); (2) resided in the target region for more than 6 months; and (3) did not have serious diseases (e.g., nephropathy, CVD, or cancer); and (21) written informed consent was obtained from participants and their parents/guardians. Exclusion criteria: incomplete information such as age, gender, nut dosage, and so on. At baseline, all the participants completed the socioeconomic status (SES) and family health history questionnaires and were recruited mainly from grade one to grade six based on primary public-school screening. The questionnaires were administered and collected by the teachers. Twenty thousand children were planned to be included, after excluding participants who refused to sign informed consent or had missing information in questionnaires at baseline (*n* = 4,732), a total of 15,268 children with complete data were included in the final analysis sample. The flowchart of inclusion/exclusion of participants is shown as Figure 1. This study was approved by the Institutional Review Board at the Children's Hospital of Chongqing Medical University (ethic approval number 2013 - 86).

### Data Collection and Diagnostic Criteria

Data on demographics were collected by trained interviewers using a structured questionnaire. Anthropometric variables of BP, height, weight, and waist circumference (22) were measured by well-trained pediatrics nurses, and the protocol for these measurements was described in a previous publication (23).

Blood pressure (BP) and heart rate were measured on three separate occasions using an OMRON arm-type electronic sphygmomanometer (HEM7051, Dalian Co. LTD, China), and the details of how measurements were performed are described in a previous publication (19, 23). Secondary hypertension was excluded by a pediatrician through an interview regarding the medical history of the subject and by performing physical examinations on hypertensive participants in stage two. The hypertension diagnostic criteria described by Mi Jie were considered suitable for the growth characteristics of children and teenagers in this study (24). Hypertension was defined as mean measured SBP and/or DBP ≥95th percentile, based on age, sex, and height percentiles. Participants were diagnosed with hypertension if all three BP measurements met the criteria for hypertension. Mean arterial pressure (MAP, mmHg) is calculated as MAP = DBP (mmHg)+ 1/3 (SBP (mmHg)− DBP (mmHg)).

### Demographic Variables, Nut Intake

Demographic variables, SES variables, prenatal variables, and dietary intake variables were collected, which were described in detail in previous publications (19, 25). Family history of nut intake was surveyed by questionnaires. Moreover, nut intake was surveyed by the self-reported food intake frequency questionnaire. A quantitative food frequency questionnaire was used to collect dietary information, which was introduced in detail in a previous publication (26). In brief, the amount of food in each category was investigated by interviewing the parents about the amount and frequency of intake during a measurable interval. Numbers were used to represent the frequency: 0 = not eating, 1 = days, 2 = weeks, 3 = months, and 4 = years. Then, the corresponding consumption amount was filled to indicate how many nuts children eat each day, week, month, or year. The total amount of food was divided by the number of days to determine the average daily intake. Standardized food and drink vessels (bowl, plate, cup, spoon, etc.) were used to display standard weights of food to ensure the validity of the survey.

### The Grouping Method of Nut

**Figure 2:**
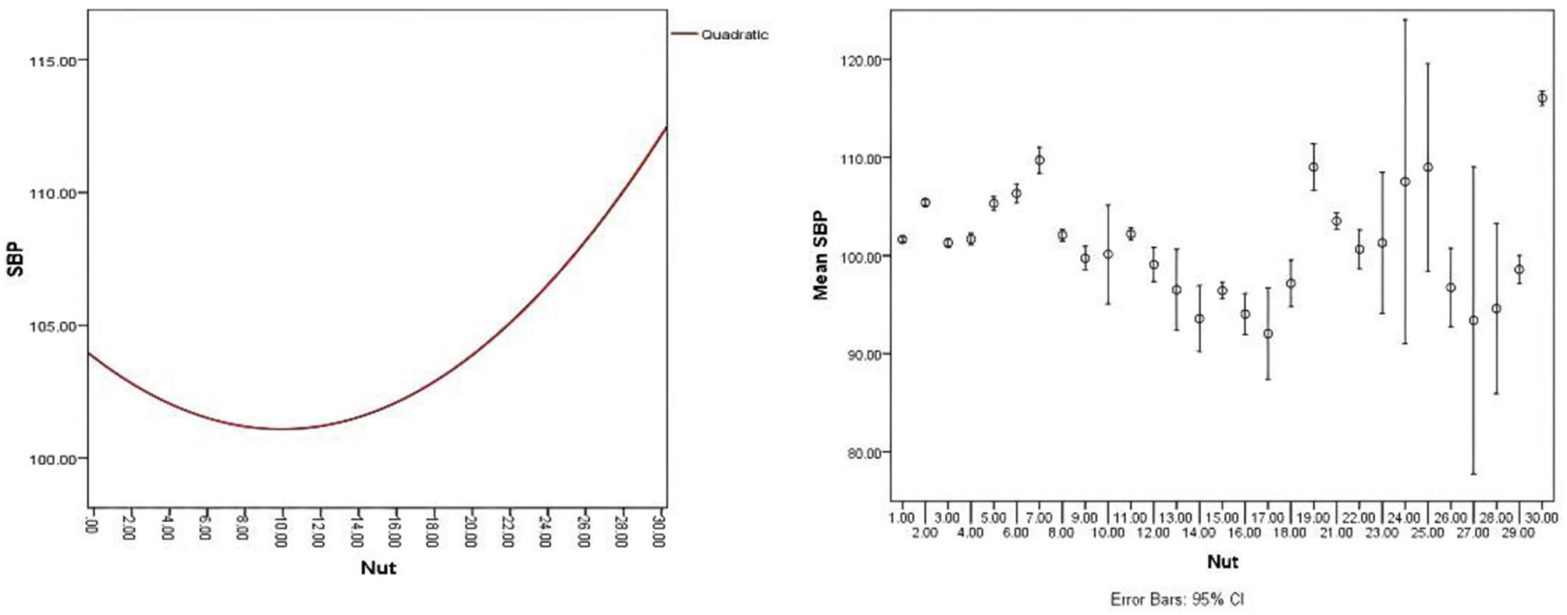
A U-shaped relationship between nuts intake and systolic blood pressure (SBP) with an error bar of 95% CI.

**Figure 3:**
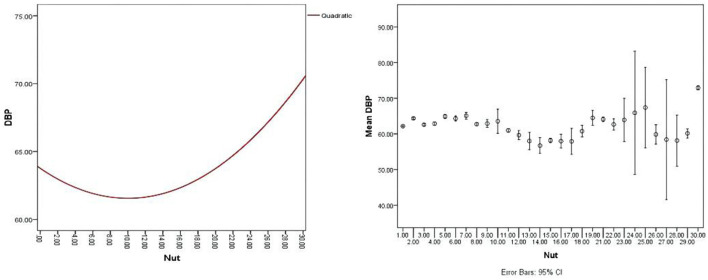
A U-shaped relationship between nuts intake and diastolic blood pressure (DBP) with an error bar of 95% CI.

**Figure 4:**
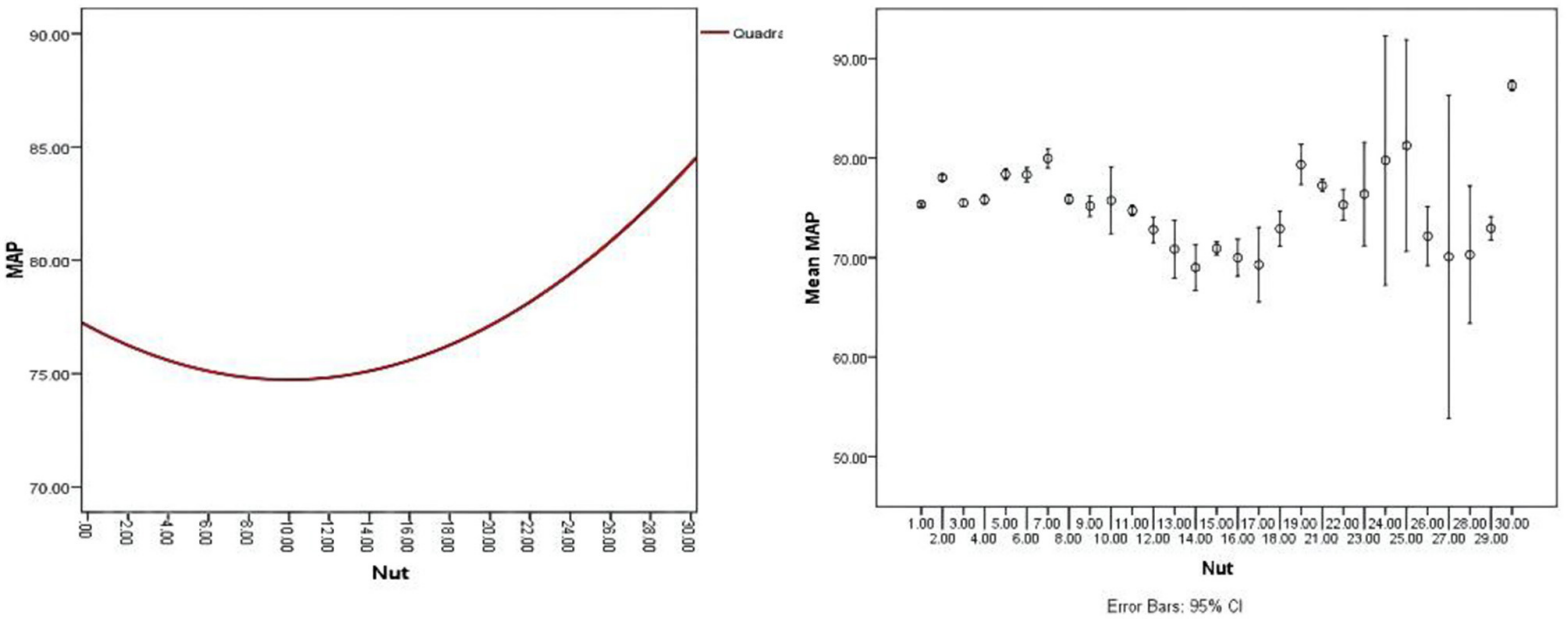
A U-shaped relationship between nuts intake and mean blood pressure (MBP) with an error bar of 95% CI.

First, the nut dosage was divided into 30 groups from 0 to 150 g by 5 g interval group, and the mean value was used to calculate the mean blood pressure (MBP) of children in each interval group. The nonlinear relationship between nuts and BP was observed by drawing a bar chart. Then, we merged similar results into 4 groups and used curve fitting using the regression model to fit the curve of correlation between BP and nuts intake. The curve revealed that a U-shaped relationship between nuts intake and BP level was observed (Supplementary Figures 1–3, Figures 2–4). We divided the daily dose of nuts into 4 groups, <35 g/day, 35 g/day ≤ nut <50 g/day, 50~100 g/day (≥50 and ≤100 g), and >100 g/day.

### Statistical Analyses

The continuous variables were reported as means and SDs if the variables meet normal distribution, and the *t*-test was used to test the significance of the difference between the two groups. Continuous variables that do not satisfy the normal distribution were expressed as X 50% (X 25%, X 75%), and the Wilcoxon rank-sum test was used for comparison between the two groups. The categorical variables were reported as numbers (*n*) and percentages of the total (%), and χ^2^ test was used to test the difference between the two groups. Moreover, the univariate logistic regression model was performed to analyze the relationship between hypertension and SES, perinatal measures, anthropometric. In addition, general linear models (GLM) were used to evaluate the relationship between BP level and nuts intake. Model 1 is a crude model, model 2 adjusted sex and age, and model 3 adjusted for all the significant factors (e.g., age, income, father occupation, mother occupation, weather breastfed, body mass index, waist circumference, gestational hypertension, gestational diabetes, physical activity in school, physical activity in home, physical activity in total, cereals and potatoes, milk, bean food, mushrooms and algae food, pickles, and beverages). Furthermore, subgroup analyses of sex were made to explore the difference of sex. Statistical significance was set at *p* < 0.05. All statistical analyses were performed using SAS 9.4 software (SAS Institute Inc., Cary, NC, USA).

## Results

### General Characteristics

Overall, 5,268 children with a mean age of 9.25 ± 1.74 years, among them, with 7,937 (54.98%) boys were included in the analysis (Table 1). The dose of nuts intake was divided into four groups according to the dose-relationship with children's BP level (Table 2). We observed statistically significant differences in several characteristics among the four nuts intake groups (Table 1). *Post-hoc* pairwise comparisons showed that there was a significant difference in SBP and mean artery pressure (27) among 4 groups (*p* < 0.001). However, there was no significant differences in DBP between the group <35 g/day and 35 g/day ≤ nut < 50 g/day (*p* > 0.05). Table 2 displayed the percentage of nuts consumed in the four groups by sex and age subgroups. Of all the children, 11,130 (72.9%) participants consumed <35 g/day of nuts, 1,145 (7.5%) participants consumed 35 g/day ≤ nut <50 g/day of nuts, 2,053 (13.4%) participants consumed 50~100 g/day of nuts, and only 940 (6.2%) consumed over 100 g/day of nuts. For the sex subgroup, 1,074 (13.53%) and 979 (13.35%) of boys and girls consumed 50~100 g/day of nuts (Table 2). The proportion of children who consumed 50~100 g/day of nuts per day was the highest in 8–9 years age groups, i.e., 876 (17.51%).

**Table 1:** General characteristics of children in this study.

**Variables**	**Total**	**Nut (g/d)**				**Overall *p***
		** <35 g/d**	**35 ≤nut <50**	**50**~**100 g/d**	**>100 g/d**	
**Sample size, [*****n*** **(%)]**	15,268	11130 (72.9)	1145 (7.5)	2053 (13.4)	940 (6.2)	
**Gender, male [*****n*** **(%)]**	7,937 (51.98)	5708 (51.28)	644 (56.24)	1074 (52.34)	511 (54.36)	
**AGE, year**	9.25 ± 1.74					
6–7	4,458 (29.20)	3,145 (28.26)	512 (44.72)	374 (18.22)	427 (45.43)	<0.001
8–9	5,003 (32.77)	3,668 (32.96)	209 (18.25)	876 (42.67)	250 (26.60)	
>10	5,807 (38.03)	4,317 (38.79)	424 (37.03)	803 (39.11)	263 (27.48)	
**BMI, kg/m2**	17.38 ± 3.06	17.27 ± 2.98 ^234^	17.49 ± 3.17 ^14^	17.53 ± 3.15 ^14^	18.20 ± 3.53 ^123^	<0.001
**Height, cm**	134.67 ± 11.44	134.59 ±1 1.42 ^234^	133.68 ± 12.96 ^13^	136.10 ± 10.42 ^124^	133.61 ± 11.55 ^13^	<0.001
**Weight, kg**	32.13 ± 9.54	31.87 ± 9.28 ^34^	32.10 ± 10.84 ^34^	32.99 ± 9.20 ^12^	33.34 ± 11.25 ^12^	<0.001
**Waist circumference, cm**	58.32 ± 8.47	57.88 ± 8.36 ^123^	58.62 ± 8.92 ^134^	59.68 ± 7.98 ^12^	60.18 ± 9.67 ^12^	<0.001
**Heart rate, n/min**	94.67 ± 12.53	94.94 ± 12.52 ^234^	92.96 ± 11.06 ^14^	92.50 ± 12.10 ^14^	98.23 ± 14.11 ^123^	<0.001
**SBP**	103.10 ± 9.94	103.00 ± 9.42 ^234^	101.75 ± 9.57 ^134^	**100.43** ±**9.80 ^124^**	111.71 ± 11.88^123^	<0.001
**DBP**	63.18 ± 7.65	63.08 ± 7.23 ^34^	62.78 ± 7.34 ^34^	**60.85** ±**7.54 ^124^**	69.91 ± 9.22 ^123^	<0.001
**MAP**	76.48 ± 7.70	76.39 ± 7.24 ^234^	75.77 ± 7.36 ^134^	**74.04** ±**7.66 ^124^**	83.84 ± 9.02 ^123^	<0.001
**Breast feeding [*****n*** **(%)]**						
NO	2,177 (14.46)	1,568 (14.30)	182 (16.05)	298 (14.65)	129 (13.99)	0.427
YES	12,881 (85.54)	9,400 (85.70)	952 (83.95)	1,736 (85.35)	793 (86.01)	
**Birth weight [*****n*** **(%)]**						
<3000	4,132 (27.17)	2,994 (27.03)	331 (29.01)	572 (27.89)	235 (25.08)	<0.001
3001 <3600	6,613 (43.49)	4,713 (42.55)	523 (45.84)	957 (46.66)	420 (44.82)	
>3600	4,461 (29.34)	3,370 (30.42)	287 (25.15)	522 (25.45)	282 (30.10)	
**Weight, kg [*****n*** **(%)]**						
Normal	4,132 (27.17)	2,994 (27.03)	331 (29.01)	572 (27.89)	235 (25.08)	<0.001
Overweight	6,613 (43.49)	4,713 (45.84)	523 (46.66)	957 (44.82)	420 (43.49)	
Obesity	4,461 (29.34)	3,370 (30.42)	287 (25.15)	522 (25.45)	282 (30.10)	
**Father with obesity [n (%)]**						
No	12,649 (83.91)	9,211 (83.92)	951 (84.16)	1,719 (84.22)	768 (82.85)	0.807
Yes	2,425 (16.09)	1,765 (16.08)	179 (15.84)	322 (15.78)	159 (17.15)	
**Mother with obesity [*****n*** **(%)]**						
No	13,580 (90.12)	9,838 (89.63)	1,051 (92.93)	1,864 (91.60)	827 (89.31)	<0.001
Yes	1,488 (9.88)	1,138 (10.37)	80 (7.07)	171 (8.40)	99 (10.69)	
**Father's occupation [*****n*** **(%)]**						
Manager	1,258 (8.36)	804 (7.35)	152 (13.39)	228 (11.15)	74 (7.95)	<0.001
Worker	4,848 (32.21)	3,343 (30.56)	426 (37.53)	790 (38.65)	289 (31.04)	
Technicist/Researcher	629 (4.18)	462 (4.22)	46 (4.05)	81 (3.96)	40 (4.30)	
Farmer	4,865 (32.33)	3,779 (34.54)	256 (22.56)	494 (24.17)	336 (36.09)	
Others	3,450 (22.92)	2,552 (23.33)	255 (22.47)	451 (22.06)	192 (20.62)	
**Mother's occupation [*****n*** **(%)]**						
Manager	850 (5.66)	524 (4.80)	114 (10.04)	164 (8.06)	48 (5.18)	<0.001
Worker	4,287 (28.54)	2,930 (26.82)	389 (34.27)	699 (34.33)	269 (29.02)	
Technicist/ Researcher	275 (1.83)	199 (1.82)	26 (2.29)	30 (1.47)	20 (2.16)	
Farmer	4,511 (30.03)	3,363 (30.78)	322 (28.37)	589 (28.93)	237 (25.57)	
Others	5,100 (33.95)	3,909 (35.78)	284 (25.02)	554 (27.21)	353 (38.08)	
**Income, Yuan/month [*****n*** **(%)]**						
<500	841 (5.55)	674 (6.11)	41 (3.62)	68 (3.33)	58 (6.20)	<0.001
<1,000	1,629 (10.75)	1,293 (11.71)	86 (7.60)	144 (7.06)	106 (10.75)	
<2,000	2,902 (19.73)	2,178 (18.20)	206 (17.44)	356 (17.31)	162 (19.16)	
<3,000	3,653 (24.12)	2,656 (28.80)	326 (25.72)	525 (25.32)	237 (24.12)	
>3000	6,122 (40.42)	4,328 (39.21)	473 (21.78)	948 (46.45)	373 (39.85)	
**Gestational diabetes [*****n*** **(%)]**						
NO	14,767 (99.31)	10,729 (99.26)	1,124 (99.73)	2,005 (99.45)	909 (99.02)	0.167
YES	103 (0.69)	80 (0.74)	3 (0.27)	11 (0.55)	9 (0.98)	
**Gestational hypertension [*****n*** **(%)]**						
NO	14,543 (98.49)	10,579 (98.53)	1,098 (98.30)	1,972 (98.55)	894 (98.13)	0.751
YES	223 (1.51)	158 (1.47)	19 (1.70)	29 (1.45)	17 (1.87)	
**Live with grandparents [*****n*** **(%)]**						
NO	12,895 (84.63)	9,432 (84.96)	952 (83.29)	1,745 (85.00)	766 (81.58)	0.024
YES	2,342 (15.37)	1,670 (15.04)	191 (16.71)	308 (15.00)	173 (18.42)	
**Medical insurance [*****n*** **(%)]**						
NO	12,775 (84.11)	9,412 (85)	897 (78.89)	1,692 (82.78)	774 (82.87)	<0.001
YES	2,413 (15.89)	1,661 (15)	240 (21.11)	352 (17.22)	160 (17.13)	
**Physical activity [*****n*** **(%)]**						
School, min						
<10	7,998 (52.66)	5,895 (53.24)	614 (53.95)	981 (48.04)	508 (54.33)	<0.001
> 10	7,190 (47.34)	5,178 (46.76)	524 (46.05)	1,061 (51.96)	427 (45.67)	
Home, min						
<55	9,813 (64.64)	7,289 (65.83)	700 (61.73)	1,232 (60.27)	592 (63.52)	<0.001
>55	5,369 (35.36)	3,783 (34.17)	434 (38.27)	812 (39.73)	340 (36.48)	
Total, min						
<150	7,846 (51.77)	5,800 (52.49)	597 (52.65)	957 (46.89)	492 (52.85)	<0.001
>150	7,309 (48.23)	5,249 (47.51)	537 (47.35)	1,084 (53.11)	439 (47.15)	
**Dietary intake, median (P25, P75) (g)**						
Cereals and potatoes	150 (100, 250)	150 (100, 250)	200 (100, 250)	177.62 (78.57, 298.21)	150 (100, 250)	0.013
Vegetables	150 (100, 250)	150 (100, 250)	200 (100, 250)	150 (100, 250)	150 (100, 250)	0.07
Fruit	150 (71.43, 250)	150 (71.43, 250)	142.86 (71.43, 250)	150 (71.43, 250)	150 (71.43, 250)	0.076
Red meat	100 (50, 150)	100 (50, 150)	100 (50, 150)	100 (50, 150)	100 (50, 150)	0.737
Poultry	35.71 (14.29, 71.43)	35.71 (14.29, 71.43)	28.57 (14.29, 51.67)	28.57 (14.29, 71.43)	28.57 (14.29, 71.43)	<0.001
Fish	16.67 (5.00, 35.71)	16.67 (5.00, 35.71)	14.29 (3.50, 35.71)	14.29 (3.33, 35.71)	14.29 (5.00, 35.71)	0.042
Eggs	50.00 (18.33,100.00)	50.00 (16.67,100.00)	50.00 (21.43,100.00)	50.00 (21.43,100.00)	50.00 (20.87,100.00)	0.53
Milk	250 (160, 260)	250 (150, 250)	250 (200, 300)	250 (200, 300)	250 (150, 300)	<0.001
Bean food	28.57 (14.29, 71.43)	28.57 (14.29, 71.43)	25.00 (8.33, 71.43)	21.43 (8.33, 71.43)	28.57 (14.29, 71.43)	0.001
Mushrooms and algae food	8.33 (1.67, 30)	8.33 (1.67, 33.33)	8.33 (1.67, 26.60)	8.33 (1.67, 28.57)	8.33 (1.67, 28.57)	0.661
Oils	50 (35.71, 100)	50 (40.00, 100)	50 (33.33, 100)	50 (35.71, 100)	50 (28.57, 100)	<0.001
Pickle	7.14 (0.33, 16.67)	7.14 (0.55, 21.43)	5.00 (0.14, 14.29)	3.57 (0, 14.29)	7.14 (0.41, 20.00)	<0.001
Nutritional supplements	0 (0, 5)	0 (0, 5.48)	0 (0, 3.33)	0 (0, 1.84)	0 (0, 5.21)	0.003
Beverage	14.29 (0.68, 50)	14.29 (0.68, 50)	13.33 (0.82, 42.86)	16.67 (1.37, 57.14)	14.29 (0.68, 42.86)	0.317

1234 *Comparisons between subgroups are significant. (p <0.05). Bold: For emphasis, this group of nuts(50-100 g/d) had the lowest SBP, DBP and MAP*.

**Table 2:** Nuts intake percentage by age and sex subgroup.

**Variables**	**Nuts (g/d)**				***p***
	** <35**	**35 ≤nut <50**	**50**~**100**	**>100**	
Sex					0.005
Male [*n* (%)]	5,708 (71.92)	644 (8.11)	1,074 (13.53)	511 (6.44)	
Female [*n* (%)]	5,422 (73.96)	501 (6.83)	979 (13.35)	429 (5.85)	
AGE, years, [*n* (%)]					<0.001
6–7	3,145 (70.55)	512 (11.48)	374 (8.39)	427 (9.58)	
8–9	3,668 (73.32)	209 (4.18)	876 (17.51)	250 (5.00)	
>10	4,317 (74.34)	424 (7.30)	803 (13.83)	263 (4.53)	

### The Association Between Nuts Intake and Hemodynamic Indexes

As shown in Table 3, the GLM model 1 to model 3 illustrated the U-shaped relationship between nuts intake and hemodynamic indexes. Compare with 50~100 g/day of nuts intake group, <35 g/day, 35 g/day ≤ nut <50 g/day, and > 100 g/day groups are associated with an increased risk of higher SBP (β = 2.573, *p* < 0.001, β = 1.317, *p* < 0.001, and β = 11.278, *p* < 0.001, respectively), DBP (β = 2.225, *p* < 0.001, β = 1.927, *p* < 0.001, and β = 9.057, *p* < 0.001, respectively), and MAP (β = 2.341, *p* < 0.001, β = 1.724, *p* < 0.001, and β = 9.797, *p* < 0.001, respectively) in model 1 (the crude model). In addition, after adjusted age and sex in model 2, or adjusted all the significant factors (such as, age, family income, parental occupation, breastfed, BMI, waistline, gestational hypertension, gestational diabetes, and dietary intake of cereals, potatoes, milk, bean food, mushrooms and algae food, pickle, and beverage) in model 3, there were similar trends about the relationship between nuts intake and SBP (β = 3.367, *p* < 0.001, β = 2.191, *p* < 0.001, and β = 11.549, *p* < 0.001, respectively), DBP (β = 2.449, *p* < 0.001, β = 2.397, *p* < 0.001, and β = 9.087, *p* < 0.001, respectively), and MAP (β = 2.755, *p* < 0.001, β = 2.329, *p* < 0.001, and β = 9.908, *p* < 0.001, respectively). As shown in Supplementary Tables 1, 2, three GLM models was constructed by sex, and the nuts and hemodynamic indicators can draw the same conclusions as the full samples.

**Table 3:** Multivariate regression analysis with systolic blood pressure (SBP), diastolic blood pressure (DBP), mean arterial pressure (MAP) as the dependent variables.

**Variables (total)**	**SBP**			**DBP**			**MAP**		
	**β**	**SE**	***P***	**β**	**SE**	***P***	**β**	**SE**	***P***
**Model 1 crude nuts (ref. 50 ~ 100 g/d)**									
Nuts (<35 g/d)	2.573	0.232	<0.001	2.225	0.178	<0.001	2.341	0.178	<0.001
Nuts (35 ≤ nut <50)	1.317	0.356	<0.001	1.927	0.273	<0.001	1.724	0.274	<0.001
Nuts (> 100 g/d)	11.278	0.38	<0.001	9.057	0.292	<0.001	9.797	0.293	<0.001
**Model 2 nuts (ref. 50** **~** **100 g/d)**									
Nuts (<35 g/d)	2.946	0.217	<0.001	2.387	0.176	<0.001	2.573	0.173	<0.001
Nuts (35 ≤ nut <50)	2.266	0.336	<0.001	2.404	0.272	<0.001	2.358	0.267	<0.001
Nuts (>100 g/d)	12.715	0.357	<0.001	9.674	0.29	<0.001	10.688	0.284	<0.001
**Model 3 nuts (ref. 50~100 g/d)** ^a^									
Nuts (<35 g/d)	3.3668	0.213	<0.001	2.449	0.183	<0.001	2.755	0.175	<0.001
Nuts (35 ≤ nut <50)	2.191	0.326	<0.001	2.397	0.28	<0.001	2.329	0.268	<0.001
Nuts (>100g)	11.549	0.349	<0.001	9.087	0.3	<0.001	9.908	0.288	<0.001

*Model 1: crude*.

*Model 2: adjusted age and sex*.

*Model 3: adjusted age, sex, income, father occupation, mother occupation, weather breastfed, body mass index (BMI), waist circumference, gestational hypertension, gestational diabetes, cereals and potatoes, milk, bean food, mushrooms and algae food, pickle, and beverage*.

a *A total of 1,430 participants having missing values in model 3*.

*Bold: For emphasis, this group of nuts(50-100 g/d) had the lowest SBP, DBP and MAP*.

### The Relationship Between Nuts Intake and Hypertension in Children

The relationship between nut intake and BP is not a simple linear. In group 50~100 g/day, the SBP (100.43 ± 9.80, Table 1) and DBP (60.85 ± 7.54, Table 1) were the lowest among the 4 groups. As for children with hypertension, the logistic regression model 1 (the crude model, Table 4) showed that compared with the 50~100 g/day group, the other three groups are more likely to have higher BP in children (odds ratio [*OR*] = 1.659, *OR* = 1.643, and *OR* = 21.368, respectively, all *p* < 0.001). In addition, for adjusted age and sex in model 2 (*OR* = 1.658, *OR* = 1.648, and *OR* = 21.413, respectively, all *p* < 0.001), or adjusted all the significant factors in model 3 (*OR* = 1.653, *OR* = 1.568, and *OR* = 19.928, respectively, all *p* < 0.001), there were similar trends to increase the risk of hypertension in children when compared with the 50~100 g/day group. Among the 4 nuts groups, the nuts intake over 100 g/day causes hypertension was far beyond other groups.

**Table 4:** The risk factors of children with hypertension in logistic regression model.

**Variables**	**β**	**SE**	***P***	**OR (95%)**
**Model 1 crude nuts (ref. 50** **~** **100 g/d)**				
Nuts (<35 g/d)	0.51	0.044	<0.001	1.659 (1.377, 1.998)
Nuts (35 g/d ≤ nut <50 g/d)	0.52	0.079	<0.001	1.643 (1.267, 2.130)
Nuts (>100 g/d)	2.046	0.06	<0.001	21.368 (17.169, 26.594)
**Model 2 nuts (ref. 50** **~** **100 g/d)**				
Nuts (<35 g/d)	0.511	0.044	<0.001	1.658 (0.965, 1.183)
Nuts (35 g/d ≤ nut <50 g/d)	0.518	0.079	<0.001	1.648 (1.270, 2.137)
Nuts (> 100 g/d)	2.047	0.061	<0.001	21.413 (17.189, 26.674)
**Model 3 nuts (ref. 50 ~ 100 g/d) ^a^**				
Nuts (<35 g/d)	0.491	0.048	<0.001	1.638 (1.341, 2.001)
Nuts (35 g/d ≤ nut <50 g/d)	0.532	0.84	<0.001	1.572 (1.189, 2.077)
Nuts (>100 g/d)	2.008	0.066	<0.001	19.934 (15.727, 25.266)

*Model 1: crude*.

*Model 2: adjusted age and sex*.

*Model 3: adjusted age, income, father occupation, mother occupation, weather breastfed, BMI, waist circumference, gestational hypertension, gestational diabetes, physical activity in school, physical activity in home, physical activity in total, cereals and potatoes, milk, bean food, mushrooms and algae food, pickle, and beverage*.

a *A total of 1,512 participants having missing values in model 3*.

## Discussion

The present study has shown that nut intake is associated with hemodynamic indexes, such as SBP, DBP, and MAP in children aged from 6 to 12 years. Our study revealed a nonlinear dose-response relationship between nut consumption and BP (Figures 2–4), and consuming nuts from 50 to 100 g/day is the most beneficial dosage for modulating childhood BP, in both boys and girls. To our knowledge, this is the first study designed to assess the effects of optimal doses of nut intake on BP level in children.

There are a wide variety of nuts, and each nut has different effects on BP. The most consumed nuts in western countries are walnuts, pistachios, cashews, almonds, and hazelnuts (28). In China, macadamia nuts, pecans, and almonds are preferred. For Chinese children, the types of their nut diet include peanuts, melon seeds, walnuts, almonds, and so on. The different types of nuts may have different effects on BP levels (12, 28–30). Regardless of daily nut type, a mixture effect on the BP has been reported (28). This is consistent with our finding that nuts have an effect on BP. It was the first study, which provided evidence of the relationship between nut intake and BP level in Chinese children.

The results about the effect of nuts on BP level remain controversial. Del Gobbo et al. (29) reported that the tree nut intake did not lower BP. However, another meta-analysis showed that nut intake might decrease SBP and DBP (31). Different types of nuts, varied dosage, and the duration of intake nuts may cause different conclusions (32). However, most studies have shown that nuts have a beneficial effect on SBP and DBP, respectively or both. In addition, the mechanisms concerning the effects of nuts on the BP of children may be different from adults.

Nuts have been reported to regulate BP in previous studies, mainly from adults. Studies demonstrated that cashew nut consumption might reduce SBP but has no effects on lipid profile and DBP (17). Moreover, a meta-analysis showed that almonds might have a considerable favorite effect in BP, especially in DBP (33). The previous study mainly focused on adults, but in this study, we added relevant evidence that nuts are associated with a decrease in both SBP and DBP in children. Participants in our study usually consider peanuts as nuts (32), although peanuts are precisely legumes, hence, “nuts” in the current article include peanuts. For Chinese children, the types of their nut diet include peanuts, melon seeds, walnuts, almonds, and so on, which are mainly peanuts and melon seeds. But the type of nut studied abroad is mainly almonds, cashews, walnuts, and peanuts (34). The different types of nuts may have different effects on BP levels. It was the first study, which provided evidence of the relationship between nut intake and BP level in Chinese children.

Several studies have explored the relationship between nut intake dosage and BP level. Mediterranean diet supplemented with 30 g of mixed nuts/day (15 g of walnuts, 7.5 g of hazelnuts, and 7.5 g of almonds) lowers BP compared with the typically recommended low-fat diet (35–37). In adults, a systematic demonstrated that taking one 1 oz serving/day nut lowered total cholesterol, low-density lipoprotein (LDL) cholesterol, Apolipoprotein B (ApoB), and triglycerides, but no statistically significant effects were found on SBP and DBP (29). The evidence from U.S. Food and Drug Administration (FDA) has indicated that eating 1.5 ounces (42 g) of tree nuts per day as part of a cholesterol and saturated fat-restricted diet might reduce the risk of CVD, but the effect on BP did not illustrate (38). The previous evidence is mostly from adults. In children, nuts were mainly part of the diet and their effect on BP and the dose-relationship between nuts and BP were not analyzed in the previous study. In addition, a U-shaped relationship between nuts intake and BP level was found in this study (Figures 2–4). The results showed that the children who intake about 50–100 g nuts daily have significantly decreased in SBP, DBP, and MAP compared with another dose intake. Moreover, the U-type relationship between nuts and BP caused us to wonder about the possible existing regulatory mechanisms.

Several mechanisms by which nuts may regulate the BP were illustrated. It was previously shown that nuts contain significant amounts of mono- and polyunsaturated fatty acids, minerals, such as magnesium, potassium and calcium, dietary fiber, and antioxidants, and all these components might interact to beneficially influence BP (17, 33, 39). Nuts may decrease BP by virtue of their low sodium and high magnesium content (40). Moreover, α-linolenic acid in walnut was related to decreased BP (30, 41), and also high MUFA intake was documented to lower BP and prevent the occurrence of cardiovascular events (17, 42). In addition, Schutte et al. found that cashew consumption is rich in PUFA, which increases baroreceptor sensitivity (BRS), and that is also beneficial for BP control (43). Eventually, the nut contains arginine, which is the precursor of nitric oxide, an endogenous vasodilator, may impact the level of BP. However, there is an increased tendency between nuts intake and BP level in children who consumed nuts more than 100 g/day compared with 50–100 g/day, but the mechanism needs to be explored in future studies.

Gender may be a key impact factor of nuts intake patterns and levels in some research. A cross-sectional study performed on 814 Australian adolescents (13–15 years old) suggests a possible role of sex in modulating the relationship between ω - 3 FA and BP: SBP and DBP were inversely associated with ω - 3 FA intake, in boys but not in girls. This difference is related to the developmental phase of the research subjects. During adolescence, the higher estrogen concentration of girls may offset the beneficial effects of dietary fatty acids (18). However, in the present study, the relationship between the changes in BP and nuts dosage in two subgroups of boys and girls were virtually the same, which could be explained by the difference of age of participants between our study (6–12 years old) and the previous study (19).

As for nut dosage, the acceptance of population and the extent to which our results are generalizable is an important consideration. In the present study, the results showed that regardless of age or sex, the number of children taking 50~100 g/day (13.4%) was relatively lower when compared with the <35 g/day (72.9%) group. To our knowledge, other studies only focused on the relationship between nuts and hemodynamic indicators in children, whereas there was no report on the comparison between daily intake nut dosage and acceptance level of children. However, a previous study demonstrated that 60 g/day of nuts significantly reduced “desire” and “liking” compared with 30 g/day, whose participants aged from 18 to 60 years old (44). The result was consistent with our findings.

The strengths of this research include three aspects. First, as far as we know, this study is the first study to explore the details of nut dosage and BP levels in children. Second, previous studies have not pointed out the relationship between highly intake dosage of nuts (>100 g/day) with BP level. This study found that the intake dosage of nuts and BP is not only a simple linear relationship. By fitting the curve, a U-shaped relationship between daily intake dosage of nuts and BP level is proposed for the first time, and the mechanisms why the high dosage of nuts can increase the BP levels are worth exploring further. At last, the results showed that an intake of 50~100 g/day nuts was more effective in affecting BP in children, but the acceptance of this daily range of dosage needs to be improved.

Our research has limitations that should be considered with interpreting the results. First, the dietary assessment relied on participants' and their parents' recall of their food intake, therefore, recall bias could not be avoided. In addition, this cross-sectional study did not divide specific types of nuts (tree nuts and peanuts), such as other research (28, 45), they did not divide specific types of nuts, and we will make supplementary inquiries about this in the future. The results showed that most children consume fewer nuts per day, which may be related to the preference of children for specific nut types. Different types of nuts and their dosage may have different effects on BP level, and further research is required in the future. Moreover, other environmental factors besides dietary intake have been considered influencing the prevalence of childhood hypertension (46, 47). However, we did not evaluate the synergistic effects between dietary intake and these factors and this analysis might be needed in the future.

In conclusion, our findings emphasize the importance that taking an appropriate amount of nuts has a beneficial effect on BP level, and indicate that an intake of 50~100 g/day nuts is the optimal daily nuts dosage of cardioprotection in children, and there is no significant sex difference on its effects. For the first time, we found a U-shaped relationship between daily nut dosage and BP level. However, most children consumed <35 g/day nuts in the present study, showing that they do not have a high acceptance of a higher daily dosage of nuts. These findings have implications for dietary advice for children. It is suggested that parents should supervise the nut intake of children, and schools can also provide nuts as a snack to ensure the daily amount of nuts intake. In the future, the types of nuts need to be refined, and in this way, giving us a better understanding of how these nuts affect the BP level, therefore it may enhance the acceptance of nuts and prevent the incidence of hypertension in children.

## Data Availability Statement

The original contributions presented in the study are included in the article/Supplementary Material, further inquiries can be directed to the corresponding authors.

## Ethics Statement

The studies involving human participants were reviewed and approved by the institutional review board at the Children's Hospital of Chongqing Medical University. Written informed consent to participate in this study was provided by the participants' legal guardian/next of kin.

## Author Contributions

XL, YB, and YF conceived and designed the study. XL and YF performed the literature search and interpretation of data. XL, XT, and XP analyzed the data. YF wrote the paper. XL, JTi, JTo, XT, and PZ performed critical revision of the manuscript for important intellectual content. All authors critically reviewed and approved the final paper.

## Funding

This work was supported by Key Development Project of Big Health of Chongqing Science and Technology Bureau (No. CSTC2021jscx-gksb-N0001), the Basic Research Project of Key Laboratory of Ministry of Education of China in 2021 (GBRP-202106), the Joint Medical Research Project of Chongqing Municipal Health Commission and Chongqing Science and Technology Bureau (No. 2020MSXM062), the Technology Foresight and Institutional Innovation Project of Chongqing Science and Technology Bureau (No. cstc2020jsyj-zzysbAX0016), the Natural Science Foundation of Youth Project (81502826), the Education Commission of Chongqing Municipality (KJQN201900443), the China Postdoctoral Science Foundation (2014M562289), and Chongqing Postdoctoral Research Funded Projects (Xm2014129).

## Conflict of Interest

The authors declare that the research was conducted in the absence of any commercial or financial relationships that could be construed as a potential conflict of interest.

## Publisher's Note

All claims expressed in this article are solely those of the authors and do not necessarily represent those of their affiliated organizations, or those of the publisher, the editors and the reviewers. Any product that may be evaluated in this article, or claim that may be made by its manufacturer, is not guaranteed or endorsed by the publisher.
