# Supplementary material for: Protective Effects of Appropriate Amount of Nuts Intake on Childhood Blood Pressure Level: A Cross-Sectional Study
Source: Front Med (Lausanne). 2022 Jan 18;8:793672. doi: 10.3389/fmed.2021.793672 (PMC8806033; doi:10.3389/fmed.2021.793672)
Supplement: Supplementary file 1 [file Table_1.DOCX]

Supplementary Material

| **[Supplementary](D:/Users/Administrator/AppData/Local/Youdao/Dict/8.10.0.0/resultui/html/index.html#/javascript:;)** **[table](D:/Users/Administrator/AppData/Local/Youdao/Dict/8.10.0.0/resultui/html/index.html#/javascript:;)** 1 Multivariate regression analysis with SBP ,DBP ,MAP as the dependent variables in male | | | | | | | | | | | |
| --- | --- | --- | --- | --- | --- | --- | --- | --- | --- | --- | --- |
| Variables | SBP | | |  | DBP | | |  | MAP | | |
|  | B | SE | P |  | B | SE | P |  | B | SE | P |
| **model 1 crude nuts(ref.50-100)** | | | | | | | | | | | |
| nuts(0-35g) | 2.314 | 0.319 | <0.001 |  | 1.968 | 0.251 | <0.001 |  | 2.083 | 0.249 | <0.001 |
| nuts(~50g) | 1.159 | 0.479 | <0.001 |  | 1.746 | 0.376 | <0.001 |  | 1.55 | 0.373 | <0.001 |
| nuts(>100g) | 11.232 | 0.516 | <0.001 |  | 8.574 | 0.405 | <0.001 |  | 9.46 | 0.402 | <0.001 |
| **model 2 nuts(ref.50-100)** | | | | | | | | | | | |
| nuts(0-35g) | 2.728 | 0.303 | <0.001 |  | 2.17 | 0.248 | <0.001 |  | 2.356 | 0.242 | <0.001 |
| nuts(~50g) | 2.282 | 0.456 | <0.001 |  | 2.277 | 0.374 | <0.001 |  | 2.279 | 0.365 | <0.001 |
| nuts(>100g) | 12.748 | 0.491 | <0.001 |  | 9.227 | 0.403 | <0.001 |  | 10.4 | 0.392 | <0.001 |
| **model 3 nuts(ref.50-100) ^a^** | | | | | | | | | | | |
| nuts(0-35g) | 3.425 | 0.295 | <0.001 |  | 2.32 | 0.259 | <0.001 |  | 2.689 | 0.246 | <0.001 |
| nuts(~50g) | 2.127 | 0.44 | <0.001 |  | 2.252 | 0.387 | <0.001 |  | 2.211 | 0.366 | <0.001 |
| nuts(>100g) | 11.576 | 0.476 | <0.001 |  | 8.66 | 0.418 | <0.001 |  | 9.632 | 0.396 | <0.001 |

Model 1: crude

Model 2: adjusted age

Model 3: adjusted age, income, father occupation, mother occupation, weather breastfed, BMI waist, gestational hypertension, gestational diabetes, cereals and potatoes, milk, bean food, mushrooms and algae food, pickle, beverage.

a. total of 813 participants having missing values in model 3

| **[Supplementary](D:/Users/Administrator/AppData/Local/Youdao/Dict/8.10.0.0/resultui/html/index.html#/javascript:;)** **[table](D:/Users/Administrator/AppData/Local/Youdao/Dict/8.10.0.0/resultui/html/index.html#/javascript:;)** 2 Multivariate regression analysis with SBP, DBP, MAP as the dependent variables in female | | | | | | | | | | | |
| --- | --- | --- | --- | --- | --- | --- | --- | --- | --- | --- | --- |
| Variables | SBP | | |  | DBP | | |  | MAP | | |
|  | B | SE | P |  | B | SE | P |  | B | SE | P |
| **model 1 crude nuts(ref.50-100)** | | | | | | | | | | | |
| nuts(0-35g) | 2.889 | 0.334 | <0.001 |  | 2.497 | 0.252 | <0.001 |  | 2.628 | 0.256 | <0.001 |
| nuts(~50g) | 1.332 | 0.529 | <0.001 |  | 2.152 | 0.399 | <0.001 |  | 1.879 | 0.405 | <0.001 |
| nuts(>100g) | 11.238 | 0.558 | <0.001 |  | 9.629 | 0.421 | <0.001 |  | 10.165 | 0.427 | <0.001 |
| **model 2 nuts(ref.50-100)** | | | | | | | | | | | |
| nuts(0-35g) | 3.17 | 0.311 | <0.001 |  | 2.621 | 0.249 | <0.001 |  | 2.804 | 0.247 | <0.001 |
| nuts(~50g) | 2.176 | 0.496 | <0.001 |  | 2.52 | 0.397 | <0.001 |  | 2.405 | 0.393 | <0.001 |
| nuts(>100g) | 12.629 | 0.522 | <0.001 |  | 10.177 | 0.418 | <0.001 |  | 10.994 | 0.413 | <0.001 |
| **model 3 nuts(ref.50-100) ^a^** | | | | | | | | | | | |
| nuts(0-35g) | 3.291 | 0.307 | <0.001 |  | 2.608 | 0.258 | <0.001 |  | 2.836 | 0.251 | <0.001 |
| nuts(~50g) | 2.254 | 0.485 | <0.001 |  | 2.565 | 0.407 | <0.001 |  | 2.461 | 0.395 | <0.001 |
| nuts(>100g) | 11.494 | 0.515 | <0.001 |  | 9.579 | 0.433 | <0.001 |  | 10.217 | 0.42 | <0.001 |

Model 1: crude

Model 2: adjusted age

Model 3: adjusted age, income, father occupation, mother occupation, weather breastfed, bmi, waist, gestational hypertension, gestational diabetes, cereals and potatoes, milk, bean food, mushrooms and algae food, pickle, beverage.

1. a total of 617 participants having missing values in model 3
