# Supplementary figures and images for: Protective Effects of Appropriate Amount of Nuts Intake on Childhood Blood Pressure Level: A Cross-Sectional Study
Source: Front Med (Lausanne). 2022 Jan 18;8:793672. doi: 10.3389/fmed.2021.793672 (PMC8806033; doi:10.3389/fmed.2021.793672)

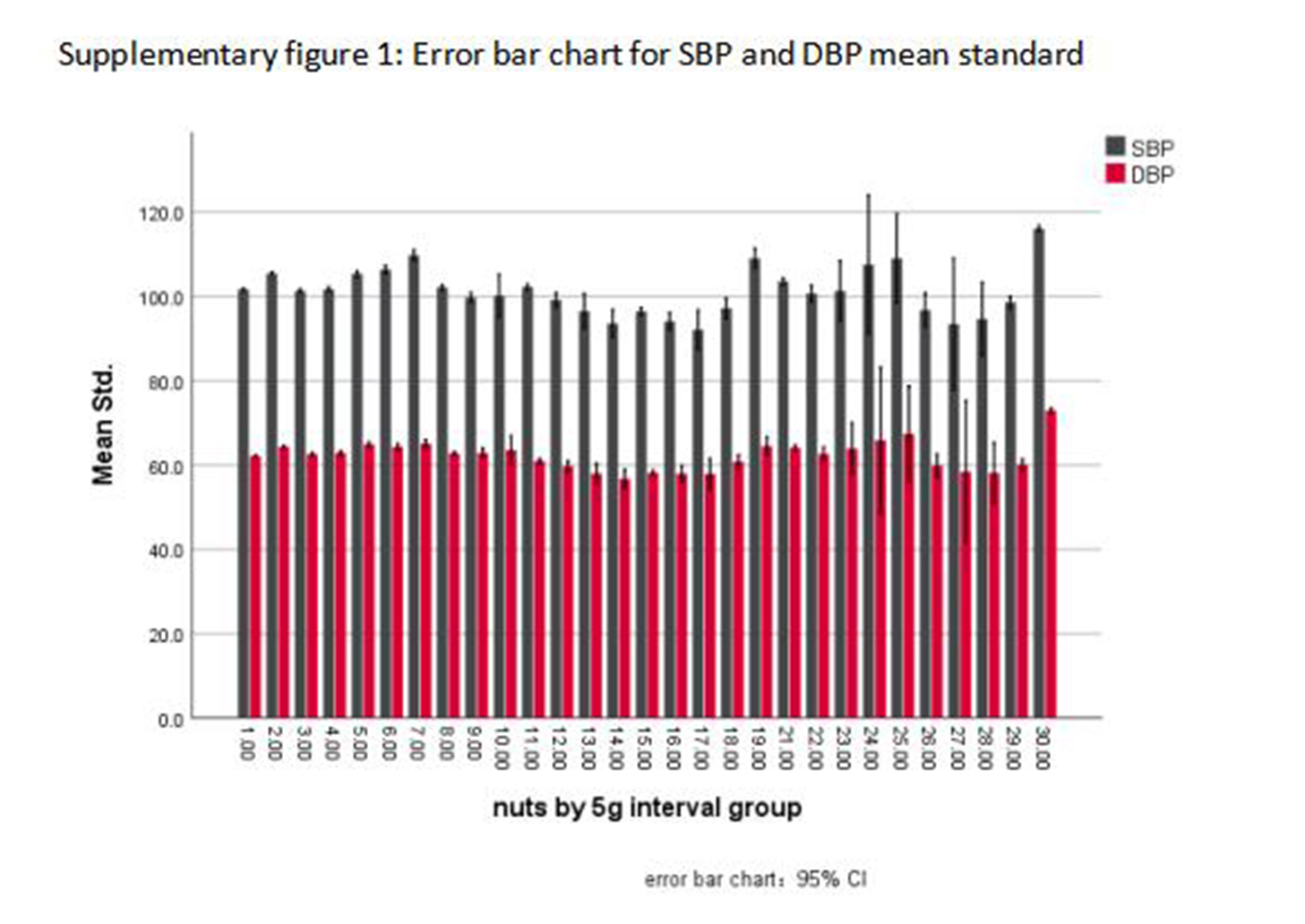

Supplement: Supplementary file 2 [file Image_1.JPEG]

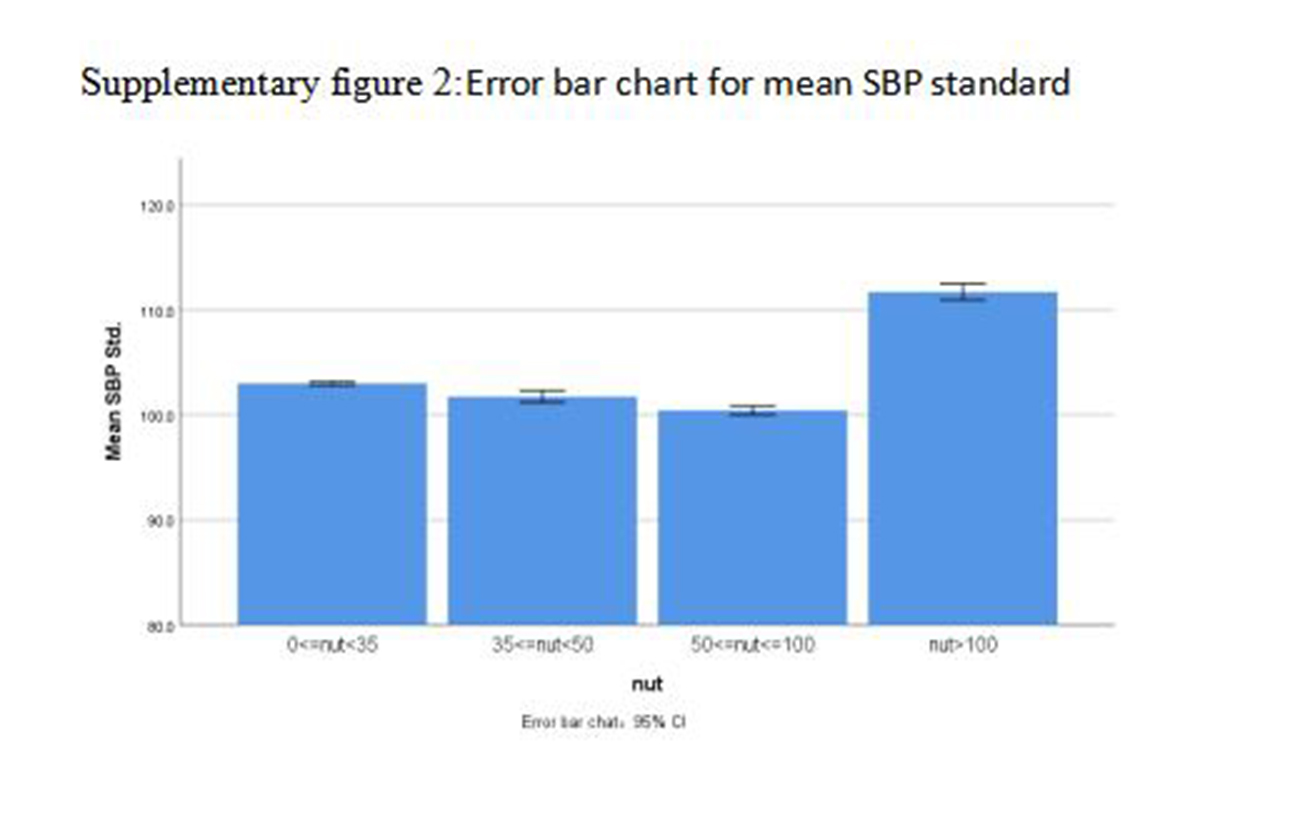

Supplement: Supplementary file 3 [file Image_2.JPEG]

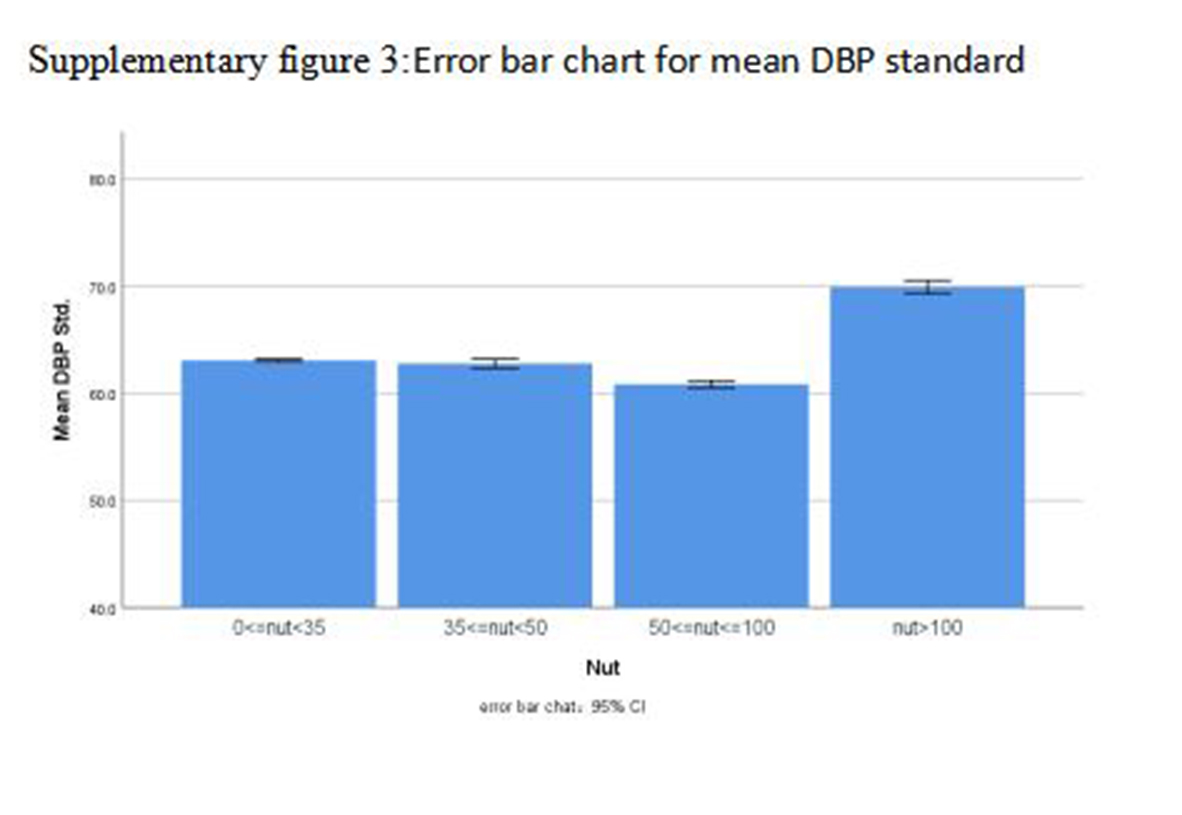

Supplement: Supplementary file 4 [file Image_3.JPEG]
